# Adaptation of the Highly Sensitive Person Scale (HSP) and Psychometric Properties of Reduced Versions of the Highly Sensitive Person Scale (R-HSP Scale) in Spanish Nursing Students

**DOI:** 10.3390/healthcare10050932

**Published:** 2022-05-18

**Authors:** Alicia Ponce-Valencia, Diana Jiménez-Rodríguez, Agustín Javier Simonelli-Muñoz, Juana Inés Gallego-Gómez, Gracia Castro-Luna, Paloma Echevarría Pérez

**Affiliations:** 1 Faculty of Nursing, Campus de los Jerónimos s/n, Catholic University of Murcia, 30107 Murcia, Spain; aponce@ucam.edu (A.P.-V.); jigallego@ucam.edu (J.I.G.-G.); pechevarria@ucam.edu (P.E.P.); 2 Department of Nursing, Physiotherapy and Medicine, University of Almeria, 04120 Almeria, Spain; sma147@ual.es (A.J.S.-M.); graciacl@ual.es (G.C.-L.)

**Keywords:** sensory processing sensitivity, environmental sensitivity, university students, nursing, scale

## Abstract

Sensory processing sensitivity (SPS) can be defined as a personality characteristic that includes the individual characteristics of sensitivity towards endogenous and exogenous stimuli. The differences in environmental sensitivity can play a crucial role in the academic context of health professionals, thus defining it as an area of research that must be addressed. The reduced scale for highly sensitive people (HSP) is a short (16 items) and adapted version of the original scale for highly sensitive people (HSP). This study aims to analyze the psychometric properties of reduced versions of the Highly Sensitive Person Scale (r-HSP Scale) in Spanish nursing students. Once the questionnaire was translated, its psychometric characteristics were analyzed. The Spanish version of the r-HSP scale was administered to 284 university students enrolled in the Nursing Degree. The results from the factorial analysis confirmed the structure of sensitiveness of six factors in our sample. This structure included the following dimensions: (1) Instability, (2) Surroundings, (3) Interaction with others, (4) Sensoperception, (5) Sensitivity, and (6) Insecurity. Additionally, the Cronbach’s α values indicated that the Spanish version of the r-HSP scale had an adequate reliability (α = 0.702). The r-HSP scale is defined as a reliable, valid, and agile replica of the original structure of sensitivity in Spanish university students.

## 1. Introduction

Sensory processing sensitivity (SPS) can be defined as a personality trait that includes the individual characteristics of sensitivity towards endogenous and exogenous stimuli [1]. It is colloquially called High Sensitivity, or Highly Sensitive Person (HSP), who are characterized by a high emotional and empathetic reactivity, and a greater depth in the processing of information [2], which makes them more vulnerable to external influences, more suggestible, and with a tendency towards sudden over-activation [3].

Sensory processing sensitivity is a non-pathological personality trait with a prevalence of 30% in the general population [4]. Initially, sensitivity was considered as a vulnerability [5]; however, recent studies have revealed adaptive traits of individuals with a high SPS, with more positive emotions in supporting environments [6,7]. In this sense, recent studies have proven that individuals with a high SPS have a greater ability to respond to positive and negative experiences [8,9]. This special sensitivity to the environment has implications on health, education, and work. Authors such as Costa-López et al. [10] and Aron et al. [3], believe that SPS is an important factor that has an influence not only on the well-being or quality of life, but also at the functional or physiological level.

It is well known that university students experience diverse stressful events [11]. Studies with samples of university students have systematically provided information on the positive correlation between SPS and symptoms of depression [12]. In the last few years, interest has also grown for studies on stress and the psychosocial factors associated with a position at work, given the repercussions they could have on the health of workers [13]. One of the groups of people that is more exposed to stress due to the characteristics of their day-to-day work is healthcare workers, with special emphasis on nursing personnel. Nevertheless, this physical and emotional overload begins previously at university, where future healthcare professionals are trained [14,15]. The sources of stress for health sciences students include balancing academic and clinical demands [16].

The 27-item Highly Sensitive Persons scale (HSP scale) was composed by Aron and Aron [1], and is the most utilized as an instrument for measuring the environmental sensitivity of students and adults. Based on this scale, others were developed for children (HSC scale) [4], as well as parents [17]. The HSP/HSC scales have been translated into various languages and their psychometric properties have been validated, although with respect to the factor structure, they revealed different solutions. Most of the results ranged from one to several structures. These authors suggested that they were closely related to neuroticism, a propensity to experience negative effects and positive openness to stimuli [18].

Sensory processing sensitivity (SPS) is a biologically based temperament trait associated with a greater awareness and ability to respond to environmental and social stimuli [19]. These individuals are characterized by being good observers and having a high creativity. However, they are introverted and can easily suffer from high levels of stress. The period at university is characterized by a greater vulnerability for a great range of mental health (MH) challenges [20], so the education context could have an important impact on the personal and professional development of students. During their university period, nursing students spend a large amount of their time in the classroom, clinical simulation rooms, and university hospitals, where they face different life experiences that can be emotionally challenging, and which can modulate their development and future well-being. In this sense, many studies consider that stress is generalized in every aspect of nursing university education [8,21,22], and that SPS increases the risk of problems related with stress as a response to negative environments [23], although it also provides a greater benefit from positive and supportive environments [24]. Therefore, the differences in environmental sensitivity play an important role in the education context, on which interventions could be made [25] to prevent the negative effects associated with SPS, and to promote its positive potential to improve the well-being and mental health of future nurses [2].

Considering the health training and education characteristics described, the differences in environmental sensitivity can play a crucial role in the academic context of health professionals, and is therefore defined as a field of research that must be addressed. For this, an abbreviated, reliable, and valid self-report is needed, that could be swiftly applied and which allows the identification of Highly Sensitive Persons among nursing students.

The general objective of the present article is to adapt the Highly Sensitive Person Scale (HSP) and to study the psychometric properties of a reduced version of the Highly Sensitive Person scale, which is widely used in Spain by the Association of Persons with High Sensitivity in Spain (Aspase), in sample of nursing students. As specific objectives, we set out to find out whether the reduced scale can be used to efficiently distinguish nursing students with HSP from those who are not, to study the prevalence, and to analyze the influence of socio-familial variables, gender and age.

## 2. Materials and Methods

### 2.1. Design

As this is an adaptation of a scale, a cross-sectional study was carried out including nursing students from the Catholic University of Murcia (UCAM, Murcia, Spain).

### 2.2. Participants

The study participants were enrolled in all four academic years in the Nursing Degree. The participants were informed about the characteristics of the study and aim of the data obtained from it. The participants provided their consent when completing the questionnaire. The final sample was composed of 284 students, enrolled in the 1st to 4th academic years within the Nursing Degree.

### 2.3. Data Collection

The study was conducted during the months of October and November, 2019. The data collection process took place during normal class hours, and the decision to participate was free and voluntary, without compensation, or disadvantages to the students who opted not to participate. Personal and academic variables were analyzed, such as gender, age, academic year, and previous healthcare education. The students were also asked whether their family, partner (if they had one at the time), and social relationships were satisfactory or unsatisfactory.

### 2.4. The Instruments for Data Colletion

Initially, the HSP scale developed by Aron and Aron (1997) was composed of 27 items. In Spain, the HSPS-S scale was validated in 2021 for an adult population, maintaining the 27 items from the original scale [26]. More recently, a reduced scale (HSC) was validated for an adolescent population [27]. Lastly, other authors at the international level also reduced the HSP scale for its use at the clinical level [28].

The values of the scale oscillate between 0 and 6 points. A higher score indicates a higher sensitivity. As a specific cut-off point does not exist for the questionnaire, the students who scored higher than the fourth quartile (4Q, ≥11 points) were defined as HSP.

### 2.5. Adaptation and Initial Validation of Instruments

For the process of translation and linguistic adaptation, the protocol suggested by Pluess [29] was followed. A committee of bilingual experts, who were educated in different disciplines, was convened. One was a physician, two were nurses, three were university professors, and two were clinical psychologists. A direct conceptual translation was made of the original in English to Spanish. Considering the cultural and university context, a provisional version was created of the reduced version of the highly sensitive person scale in Spanish, which was reviewed by a third expert. Of the 27 items from the original scale, 16 were kept in the r-HSP, as they had an item–total correlation coefficient > 0.30, considered useful for assessing the attribute under study, and a Cronbach’s α value > 0.700. Items not fulfilling this condition were excluded. Lastly, cognitive interviews were given to 10 students. The interviewees did not have any difficulties with the answer alternatives, and their general assessment of the instrument was positive. None of the interviewees manifested having comprehension problems, or mentioned the need to include other elements. The transcultural adaptation of the original version of the HSP for a Spanish population (r-HSP) had a high degree of linguistic, cultural, and conceptual equivalence.

### 2.6. Statistical Analysis

The Kolmogorov–Smirnov test was utilized to confirm the normal distribution of the continuous data, with the result being <0.05, indicating that the data did not follow a normal distribution.

To analyze the reliability of the scale, a test–retest method was applied, with the calculation of the intraclass correlation coefficient (ICC) to evaluate the degree of consistency between the quantitative measurements obtained in the questionnaire. To examine the internal consistency, Cronbach’s α was utilized, with a minimum value of 0.700 desired.

An exploratory factorial analysis (EFA) was performed. Before this, the Kaiser–Meyer–Olkin (KMO) and Bartlett’s sphericity tests were performed to consider the adjustment of the values for the EFA. So that the factorial loads were consistent, the value had to be ≥0.40 for an item to be part of the factor selected [30].

Spearman’s correlation coefficient, Welch’s *t*-test and Welch’s ANOVA were utilized. Values of *p* < 0.05 were considered significant. For the statistical analysis, the SPSS v21 software for Windows was utilized (SPSS, Inc., Chicago, IL, USA).

### 2.7. Ethical Considerations

Permission to use the English version of the 27-item standard research version was obtained via e-mail from Dr. Arthur Aron. The study was approved by the Ethics Board from the UCAM in June, 2019 (code CE 061902), considering the guidelines from the 1964 Declaration of Helsinki.

## 3. Results

Of the 284 students, 75% were women; 28.9% were enrolled in their first year, 25.4% in their second, 25% in their third, and lastly, 20.8% in their fourth year. The mean age was 21.6 ± 4.4 years. As for their training, 25.4% had some type of healthcare training. With respect to their family relationships, 8.8% described them as unsatisfactory. Additionally, 51.4% did not have a partner, and of those who did, 3.9% qualified their relationship as unsatisfactory. Lastly, 3.2% qualified their social relationships as very unsatisfactory.

### Initial Validation of the Reduced Versions of the Highly Sensitive Person Scale (R-HSP Scale)

To verify the reliability of the scale, the consistency of the items was analyzed after repeating their measurement, through the application of the intraclass correlation coefficient. Table 1 shows the ICC value of all the items in the scale, with all of them being statistically significant, with a *p* < 0.005 value.

The reliability was also verified with the correlation analysis of the different measurements obtained after applying the scale multiple times, a procedure known as the split-halves method. Thus, in Table 1 we can verify a Spearman–Brown coefficient of r = 0.886, which indicates the high reliability of the questionnaire. Additionally, the Cronbach’s α value was 0.705 on the initial test, and 0.760 on the retest, both of which were above 0.700, which verifies the reliability of the questionnaire (Table 1).

Table 2 shows the results from the correlation analysis of all the items on the questionnaire. Table 3 shows the results obtained in the homogeneity analysis of the items in the questionnaire. The Cronbach’s α value obtained was 0.702. No items were eliminated, as the Cronbach’s α value barely increased (Table 3).

As previously mentioned, the scale is composed of 16 items, with range in values between 0 and 16, with a higher score indicating a higher sensitivity. The mean was 9 ± 3.1 points, with 34.5% of the participants being HSP.

**Figure 1:**
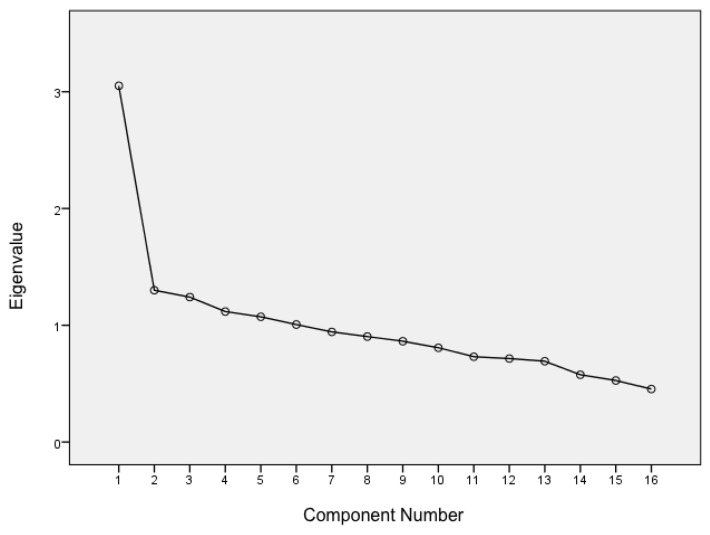
Scree Plot.

To analyze the validity of the construct, a factorial analysis was performed (Table 4). The Kaiser–Meyer–Olkin test provided a value of 0.729, with the Bartlett sphericity test being statistically significant, *p* < 0.001. The factorial analysis showed a structure composed of six factors, which as a set, explained up to 54.9% of the total variance of the results. Factor 1 with a value of 19.1%, factor 2 with 8.1%, factor 3 with 7.7%, factor 4 with 7%, factor 5 with 6.7%, and factor 6 with a value of 6.3%. Factor 1 included items 11, 12, 13, 14, and 16, which were considered related with “Instability”. Factor 2 consisted of items 3, 9, 12, and 15, related with “Surroundings”. Factor 3 was composed of items 1, 8, 10, and 13, “Interaction with others”. Factor 4 was composed of items 5 and 6, “Sensoperception”. Factor 5 included items 2 and 4, “Sensitivity”. Lastly, factor 6 was composed of items 7 and 14, “Insecurity” (Table 4). Figure 1 provides a scree plot as a graphical representation of the extracted factors.

With respect to the associations between the total HSP scale and the personal and academic factors of the university students, differences were only found in women (9.61 ± 2.99 vs. 7.1 ± 2.71; *p* < 0.001), and those who indicated having unsatisfactory family relations (10.16 ± 2.92 vs. 8.89 ± 3.10; *p* = 0.049) (Table 5).

## 4. Discussion

To measure environmental sensitivity, the most utilized scale with university students or adults is the High Sensitivity Persons scale (HSP scale) developed by Aron and Aron [1]. However, for field studies in which time is highly prized, this original version of 27 items is inconvenient due to its length, and a need was detected to validate a Reduced High Sensitivity Persons scale (r-HSP) for nursing university students. This scale included the items that were habitually used in Spain by the Association of Persons with High Sensitivity (Aspase) for the diagnosis of Environmental Sensitivity, but it is necessary to show that the r-HSP is a simple tool that can be used to identify students who are highly sensitive.

The results showed that the tool had good psychometric characteristics. More specifically, the test and the retest showed a good reliability, with Cronbach’s α values >0.700. Many studies related with the HSP scale showed one to three factors [1,28,31]. In the present study, six factors were identified. This structural model of the reduced HSP scale suggests that the general sensitivity score, as well as the scores of the six factors, are adequate for measuring the environmental sensitivity of Nursing students.

Aron and Aron [1] estimated that a high sensitivity was present in 20% of the general population. However, in the present study, 34.5% were identified, a value that is much higher than the one mentioned previously. In our study, we found significant differences, with women being much more sensitive than men. This finding is similar to the results from other authors. However, in these studies, the differences between gender groups were not statistically significant [4,32,33].

On the other hand, age was correlated with three of the six factors. The older the student, the fewer interactions with the rest, and more sensoperception and sensitivity. Costa-López et al. also found positive correlations between age and environmental sensitivity [27].

It has been described that individuals with a high level of environmental sensitivity can show over-stimulation, sensorial sensitivity, deep cognitive processing, and emotional reactivity [34]. In their social and personal relations, they are characterized as being empathetic and intuitive [3]. This means that these individuals relate better with others. These characteristics were not observed in our study, as a higher score in the scale was observed in those with unsatisfactory family relations.

Our study provides new evidence on the association between HSP and important aspects of the students, which could be considered as current life stressors, such as their relationships with their families and/or partner. The results show that those who had unsatisfactory family relations were HSP with higher scores on the scale. These results are similar to other studies, which verified that family problems of students increased their level of stress [21,22]. In other studies on HSP, research was not performed on current personal life aspects, and which directly influence their well-being. In general terms, the differential susceptibility of the adult subjects was not analyzed, including items in the questionnaires that were focused on the analysis of their childhood.

In summary, although the study used a small sample, the test–retest reliability showed ICC values (Table 1) ranging from poor (item 14, 15) to moderate and good. Furthermore, although the correlations between items were generally low, the reliability of the scale (Cronbach’s alpha in Table 3) was acceptable.

### Limitations

Just as the original scale, most of the items put emphasis on the negative traits (“I become overwhelmed when I have a lot of things to do and little time”). In future studies, it would be interesting to focus on the advantageous aspects of being an HSP, and to conduct a more in-depth analysis of other aspects such as processing ability, empathy, the emotional response ability, and the sensitivity to subtle aspects. Additionally, it would be positive to perform a multi-center study and broaden the sample to other university faculties. Due to the preliminary nature of this research, future studies are needed to confirm the results with a confirmatory factor analysis (CFA).

## 5. Conclusions

The adaptation of the reduced versions of the Highly Sensitive Person (r-HSP) scale is defined as a reliable, valid, and agile replica of the original structure of sensitivity in Spanish university students. The present initial validation of the reduced HSP scale is adequate for its application to university students, as it can distinguish between the HSP students and those who are not. The prevalence was found to be greater than the general population.

## Figures and Tables

**Table 1:** Reliability test of the questionnaire in the pilot test study.

Questionnaire Items	ICC (CI 95%)	F (*p*)
Item 1 The behavior of others affects me.	0.735 (0.655–0.796)	3.767 (<0.001)
Item 2 I tend to be sensitive to pain.	0.719 (0.634–0.784)	3.554 (<0.001)
Item 3 On busy days, I tend to need to leave, to lay down in bed, and to look for a dark room or any other place where I can find peace and relief from stimulation.	0.569 (0.439–0.668)	2.318 (<0.001)
Item 4 I am particularly sensitive to the effects of caffeine.	0.811 (0.754–0.855)	5.290 (<0.001)
Item 5 I tend to be easily overwhelmed by things such as bright lights, strong odors, coarse fabrics, or police or ambulance sirens.	0.668 (0.568–0.745)	3.014 (<0.001)
Item 6 Loud noises make me feel uncomfortable.	0.720 (0.636–0.785)	3.569 (<0.001)
Item 7 I have a rich and complex inner life, I tend to overthink things.	0.527 (0.384–0.636)	2.113 (<0.001)
Item 8 I get scared easily.	0.813 (0.756–0.856)	5.339 (<0.001)
Item 9 I become overwhelmed when I have a lot of things to do and little time	0.723 (0.640–0.787)	3.610 (<0.001)
Item 10 I am bothered that the rest want me to do too many things at the same time.	0.564 (0.433–0.665)	2.296 (<0.001)
Item 11 I tend to avoid violent films and violent series on television.	0.788 (0.724–0.837)	4.714 (<0.001)
Item 12 The activation provoked by the hustle around me is unpleasant for me.	0.518 (0.373–0.629)	2.074 (<0.001)
Item 13 Life changes shock me (moving, work changes, separations, births, deaths…).	0.680 (0.584–0.754)	3.126 (<0.001)
Item 14 When I was a child, my parents or teachers tended to see me as a sensitive or shy person.	0.314 (0.107–0.472)	1.457 (=0.003)
Item 15 For me, it is important to have a life in which I can avoid perturbing or overwhelming situations.	0.357 (0.163–0.506)	1.555 (=0.001)
Item 16 When I have to compete or be observed as I perform a task, I become nervous and unsure, and I do it worse than what I could do.	0.531 (0.389–0.639)	2.131 (<0.001)
**Split-halves analysis**		
**Cronbach’s α**		
**Test**	0.705	
**Retest**	0.760	
**Correlation between parts**	0.795	
**Spearman’s Brown coefficient**	0.886	

F: Statistic’s value. *p*: Significance level. ICC: Intraclass correlation coefficient. CI: Confidence interval.

**Table 2:** Correlation among the different questionnaire items.

ITEMS		Item 1	Item 2	Item 3	Item 4	Item 5	Item 6	Item 7	Item 8	Item 9	Item 10	Item 11	Item 12	Item 13	Item 14	Item 15	Item 16
Item 1 The behavior of others affects me.	rho *p*	-															
Item 2 I tend to be sensitive to pain.	rho *p*	0.162 0.015	-														
Item 3 On busy days, I tend to need to leave, to lay down in bed, and to look for a dark room or any other place where I can find peace and relief from stimulation.	rho *p*	0.042 0.536	0.053 0.428	-													
Item 4 I am particularly sensitive to the effects of caffeine.	rho *p*	0.062 0.360	0.165 0.013	0.027 0.684	-												
Item 5 I tend to be easily overwhelmed by things such as bright lights, strong odors, coarse fabrics, or police or ambulance sirens.	rho *p*	0.183 0.006	0.053 0.430	0.034 0.616	0.191 0.004	-											
Item 6 Loud noises make me feel uncomfortable.	rho *p*	0.129 0.053	0.210 0.002	0.156 0.019	0.147 0.028	0.456 <0.001	-										
Item 7 I have a rich and complex inner life, I tend to overthink things.	rho *p*	0.165 0.013	−0.024 0.724	−0.019 0.780	0.095 0.156	0.131 0.051	0.068 0.311	-									
Item 8 I get scared easily.	rho *p*	0.120 0.073	0.111 0.097	0.104 0.121	0.034 0.612	0.192 0.004	0.137 0.411	0.081 0.226	-								
Item 9 I become overwhelmed when I have a lot of things to do and little time	rho *p*	0.103 0.125	0.074 0.269	0.191 0.004	−0.014 0.836	0.050 0.460	0.196 0.003	0.037 0.587	0.172 0.010	-							
Item 10 I am bothered that the rest want me to do too many things at the same time.	rho *p*	0.158 0.018	0.007 0.912	0.023 0.737	0.007 0.991	0.110 0.100	0.133 0.047	0.073 0.279	0.077 0.250	0.087 0.193	-						
Item 11 I tend to avoid violent films and violent series on television.	rho *p*	0.012 0.855	0.069 0.306	−0.005 0.935	−0.021 0.754	0.118 0.079	−0.033 0.623	−0.028 0.674	0.049 0.469	−0.068 0.310	0.114 0.088	-					
Item 12 The activation provoked by the hustle around me is unpleasant for me.	rho *p*	0.008 0.990	0.185 0.005	0.070 0.298	0.103 0.124	0.261 <0.001	0.127 0.058	0.068 0.308	0.173 0.009	0.132 0.049	0.101 0.133	0.126 0.059	-				
Item 13 Life changes shock me (moving, work changes, separations, births, deaths…).	rho *p*	0.034 0.613	0.087 0.193	0.218 0.001	−0.023 0.736	0.134 0.045	0.289 <0.001	0.045 0.506	0.053 0.433	0.295 <0.001	0.081 0.227	0.059 0.380	0.255 <0.001	-			
Item 14 When I was a child, my parents or teachers tended to see me as a sensitive or shy person.	rho *p*	0.180 0.007	0.092 0.168	−0.025 0.709	0.056 0.403	0.137 0.041	0.156 0.019	0.030 0.651	0.249 <0.001	0.171 0.010	0.214 0.001	0.125 0.063	0.218 0.001	0.138 0.039	-		
Item 15 For me, it is important to have a life in which I can avoid perturbing or overwhelming situations.	rho *p*	0.107 0.110	0.181 0.007	0.149 0.026	−0.007 0.913	0.124 0.065	0.205 0.002	0.194 0.004	0.166 0.013	0.210 0.002	0.196 0.003	0.127 0.058	0.146 0.029	0.271 <0.001	0.192 0.004	-	
Item 16 When I have to compete or be observed as I perform a task, I become nervous and unsure, and I do it worse than what I could do.	rho *p*	0.059 0.381	0.173 0.010	0.140 0.037	0.161 0.016	0.153 0.022	0.242 <0.001	0.171 0.011	0.096 0.154	0.243 <0.001	0.149 0.026	0.006 0.930	0.241 <0.001	0.172 0.010	0.214 0.001	0.196 0.003	-

rho: Spearman’s correlation coefficient. *p*: Significance level.

**Table 3:** Homogeneity analysis of the questionnaire.

Questionnaire Items	Mean ± Standard Deviation	Correlation of the Items with the Total Corrected Scale	Cronbach’s α When an Item Is Eliminated
Item 1 The behavior of others affects me.	0.84 ± 0.36	0.285	0.690
Item 2 I tend to be sensitive to pain.	0.43 ± 0.49	0.276	0.691
Item 3 On busy days, I tend to need to leave, to lay down in bed, and to look for a dark room or any other place where I can find peace and relief from stimulation.	0.68 ± 0.46	0.178	0.702
Item 4 I am particularly sensitive to the effects of caffeine.	0.27 ± 0.44	0.157	0.703
Item 5 I tend to be easily overwhelmed by things such as bright lights, strong odors, coarse fabrics, or police or ambulance sirens.	0.23 ± 0.42	0.314	0.686
Item 6 Loud noises make me feel uncomfortable.	0.51 ± 0.50	0.437	0.670
Item 7 I have a rich and complex inner life, I tend to overthink things.	0.83 ± 0.37	0.185	0.699
Item 8 I get scared easily.	0.44 ± 0.49	0.275	0.691
Item 9 I become overwhelmed when I have a lot of things to do and little time	0.80 ± 0.40	0.380	0.680
Item 10 I am bothered that the rest want me to do too many things at the same time.	0.58 ± 0.49	0.229	0.696
Item 11 I tend to avoid violent films and violent series on television.	0.28 ± 0.45	0.296	0.688
Item 12 The activation provoked by the hustle around me is unpleasant for me.	0.47 ± 0.50	0.327	0.684
Item 13 Life changes shock me (moving, work changes, separations, births, deaths…).	0.75 ± 0.43	0.351	0.682
Item 14 When I was a child, my parents or teachers tended to see me as a sensitive or shy person.	0.50 ± 0.50	0.297	0.688
Item 15 For me, it is important to have a life in which I can avoid perturbing or overwhelming situations.	0.83 ± 0.37	0.377	0.681
Item 16 When I have to compete or be observed as I perform a task, I become nervous and unsure, and I do it worse than what I could do.	0.58 ± 0.49	0.421	0.672
**Cronbach’s α**		0.702	

**Table 4:** Factor loading of the questionnaire items. Rotated components matrix.

Kaiser–Meyer–Olkin Test Bartlett Sphericity Test	0.729 <0.001					
ITEMS	Factor 1 Instability	Factor 2 Environment	Factor 3 Interaction with Others	Factor 4 Sensoperception	Factor 5 Sensitivity	Factor 6 Insecurity
Item 1 The behavior of others affects me.	−0.101	0.188	0.536	−0.040	0.228	0.361
Item 2 I tend to be sensitive to pain.	0.154	0.244	0.157	0.002	0.690	−0.108
Item 3 On busy days, I tend to need to leave, to lay down in bed, and to look for a dark room or any other place where I can find peace and relief from stimulation.	−0.120	0.738	0.006	0.010	0.110	−0.095
Item 4 I am particularly sensitive to the effects of caffeine.	−0.004	−0.136	−0.004	0.244	0.667	0.158
Item 5 I tend to be easily overwhelmed by things such as bright lights, strong odors, coarse fabrics, or police or ambulance sirens.	0.006	−0.050	0.170	0.835	0.094	0.053
Item 6 Loud noises make me feel uncomfortable.	0.260	0.221	0.031	0.672	0.118	0.101
Item 7 I have a rich and complex inner life, I tend to overthink things.	0.009	0.013	0.010	0.141	0.005	0.828
Item 8 I get scared easily.	0.052	0.054	0.607	0.136	0.103	−0.057
Item 9 I become overwhelmed when I have a lot of things to do and little time	0.345	0.501	0.279	−0.099	0.042	0.065
Item 10 I am bothered that the rest want me to do too many things at the same time.	0.008	0.155	0.538	0.214	−0.368	0.131
Item 11 I tend to avoid violent films and violent series on television.	0.610	−0.096	0.126	0.258	0.068	−0.204
Item 12 The activation provoked by the hustle around me is unpleasant for me.	0.564	0.444	−0.160	0.245	−0.145	−0.080
Item 13 Life changes shock me (moving, work changes, separations, births, deaths…).	0.465	−0.025	0.589	−0.034	0.045	−0.074
Item 14 When I was a child, my parents or teachers tended to see me as a sensitive or shy person.	0.598	−0.022	0.080	−0.107	0.055	0.428
Item 15 For me, it is important to have a life in which I can avoid perturbing or overwhelming situations.	0.119	0.527	0.205	0.199	−0.093	0.249
Item 16 When I have to compete or be observed as I perform a task, I become nervous and unsure, and I do it worse than what I could do.	0.454	0.265	0.056	0.138	0.273	0.194
**Eigenvalue** **Variance**	3.051 19.1%	1.300 8.1%	1.241 7.7%	1.118 7%	1.073 6.7%	1.006 6.3%

**Table 5:** Association between each factor of the R-HSP scale and personal attribute of Spanish university students.

Variables Investigated	Factor 1	Factor 2	Factor 3	Factor 4	Factor 5	Factor 6	Total Scala HSP
Gender							
Female (*n* = 213)	2.79 ± 1.43	2.92 ± 1.03	2.82 ± 1.02	0.80 ± 0.79	0.72 ± 0.70	1.38 ± 0.65	9.61 ± 2.99
Male (*n* = 71)	1.94 ± 1.19	2.32 ± 1.22	1.94 ± 1.06	0.56 ± 0.69	0.62 ± 0.76	1.2 ± 0.68	7.1 ± 2.71
	*p* < 0.001	*p* < 0.001	*p* < 0.001	*p* = 0.027	*p* = 0.298	*p* = 0.052	*p* < 0.001
Academic year							
First (*n* = 82)	2.54 ± 1.44	2.80 ± 1.12	2.50 ± 1.25	0.65 ± 0.70	0.61 ± 0.68	1.30 ± 0.60	8.73 ± 3.19
Second (*n* = 72)	2.81 ± 1.49	2.81 ± 1.17	2.69 ± 1.03	0.78 ± 0.79	0.69 ± 0.70	1.40 ± 0.72	9.31 ± 3.42
Third (*n* = 71)	2.51 ± 1.29	2.72 ± 1.04	2.72 ± 0.97	0.75 ± 0.82	0.76 ± 0.74	1.38 ± 0.63	9.09 ± 2.59
Fourth (*n* = 59)	2.46 ± 1.47	2.76 ± 1.11	2.49 ± 1.10	0.81 ± 0.79	0.75 ± 0.77	1.22 ± 0.69	8.89 ± 3.18
	*p* = 0.479	*p* = 0.959	*p* = 0.458	*p* = 0.596	*p* = 0.572	*p* = 0.401	*p* = 0.683
Healthcare training							
No (*n* = 212)	2.58 ± 1.43	2.81 ± 1.11	2.67 ±1.08	0.66 ± 0.74	0.68 ± 0.71	1.34 ± 0.65	9.01 ± 3.08
Yes (*n* = 72)	2.60 ± 1.42	2.67 ± 1.10	2.42 ± 1.13	0.99 ± 0.81	0.75 ± 0.74	1.31 ± 0.72	8.98 ± 3.19
	*p* = 0.911	*p* = 0.339	*p* = 0.107	*p* = 0.003	*p* = 0.484	*p* = 0.724	*p* = 0.948
Family relations							
Satisfactory (*n* = 259)	2.57 ± 1.42	2.75 ± 1.12	2.58 ± 1.09	0.71 ± 0.77	0.68 ± 0.71	1.33 ± 0.68	8.89 ± 3.10
Unsatisfactory (*n* = 25)	2.68 ± 1.52	3.08 ± 0.90	2.88 ± 1.09	1 ± 0.81	0.88 ± 0.78	1.32 ± 0.55	10.16 ± 2.92
	*p* = 0.734	*p* = 0.151	*p* = 0.194	*p* = 0.104	*p* = 0.227	*p* = 0.920	*p* = 0.049
Relationship with partner							
No partner (*n* = 146)	2.58 ± 1.41	2.73 ± 1.09	2.57 ± 1.10	0.78 ± 0.77	0.71 ± 0.72	1.37 ± 0.65	9.03 ± 3.14
Satisfactory (*n* = 127)	2.64 ± 1.47	2.82 ± 1.14	2.61 ± 1.11	0.69 ± 0.77	0.68 ± 0.72	1.31 ± 0.68	8.97 ± 3.17
Unsatisfactory (*n* = 11)	2 ± 1	2.91 ± 1.04	2.91 ± 0.83	0.82 ± 0.87	0.73 ± 0.78	1.09 ± 0.70	9 ± 1.78
	*p* = 0.365	*p* = 0.727	*p* = 0.605	*p* = 0.564	*p* = 0.914	*p* = 0.357	*p* = 0.988
Social relations							
Satisfactory (*n* = 275)	2.59 ± 1.42	2.78 ± 1.10	2.59 ± 1.10	0.72 ± 0.76	0.69 ± 0.72	1.32 ± 0.67	8.97 ± 3.09
Unsatisfactory (*n* = 9)	2.33 ± 1.73	2.67 ± 1.50	3 ± 0.86	1.44 ± 0.88	0.78 ± 0.83	1.56 ± 0.52	10.11 ± 3.55
	*p* = 0.672	*p* = 0.830	*p* = 0.199	*p* = 0.005	*p* = 0.774	*p* = 0.231	*p* = 0.368
Age	rho = −0.055	rho = 0.062	rho = −0.129	rho = 0.172	rho = 0.159	rho = −0.026	rho = −0.008
	*p* = 0.352	*p* = 0.298	*p* = 0.030	*p* = 0.004	*p* = 0.007	*p* = 0.666	*p* = 0.899

rho: Spearman’s correlation coefficient. *p*: statistical significance.

## Data Availability

The data presented in this study are available on request from the corresponding author.
